# Enrichment and Portable Fiber Optic Reflectance Spectroscopy Determination of Cr(VI) Using Peroxotungstate-Anchored Water-Soluble Polymer Composites: Synthesis and Characterization

**DOI:** 10.1021/acsomega.6c01460

**Published:** 2026-04-09

**Authors:** Asiye Aslıhan Avan, Hayati Filik

**Affiliations:** Istanbul University-Cerrahpaşa, Faculty of Engineering, Department of Chemistry, Avcılar, Istanbul 34320, Turkey

## Abstract

Poly­(diallyl dimethylammonium)/peroxotungstic acid nanoparticles (PDDA@PWA NPs) were successfully synthesized via electrostatic self-assembly between the cationic polyelectrolyte PDDA and the anionic PWA species. The morphology, architecture, and composition of the PDDA@PWA NPs were systematically characterized by using transmission electron microscopy (TEM), scanning electron microscopy (SEM), Fourier transform infrared spectroscopy (FTIR), X-ray diffraction (XRD), and X-ray photoelectron spectroscopy (XPS). The newly developed, white composite exhibited excellent selectivity and adsorption capacity when used as a solid-phase extraction (SPE) platform for the efficient separation of trace Cr­(VI) ions from aqueous matrices. A portable fiber-optic reflectance spectroscopy (FORS) analysis was subsequently performed directly on the Cr­(VI)-PDDA@PWA surface employing the diphenyl carbazide (DPC) reagent. Meanwhile, the Cr­(VI)-PDDA@PWA platform displayed a color change from yellow to red-violet for trace levels of Cr­(VI) ions as low as 0.035 μM. Under the optimized FORS conditions, a linear calibration curve was obtained for Cr­(VI) concentrations ranging from 0.2 to 10 μM, based on the measured reflectance intensity of the DPC–Cr­(VI) complex. For comparative analysis and method validation, the colored DPC–Cr­(VI) complex was eluted from the nanoparticle surface using an Liebermann-Burchard reagent@N,N-dimethylformamide (LBR@DMF) solution. The absorbance of Cr­(VI) was proportional in a concentration range of 0.2–6.0 μM with a detection limit of 0.01 μM. The synthesized PDDA@PWA NPs displayed excellent FORS sensing performance for the sensitive, selective, and on-site detection of Cr­(VI) ions. The proposed approach was employed for Cr­(VI) detection in real samples, yielding satisfactory apparent recovery rates (93–97%).

## Graphical abstract

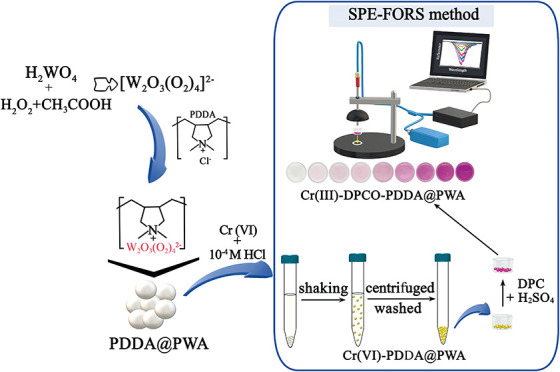

## 1 Introduction

Chromium (Cr) is a trace element whose effects on human health depend strongly on its oxidation state. Chromium exhibits nine possible valence states, ranging from −2 to +6; however, only trivalent chromium [Cr­(III)] and hexavalent chromium [Cr­(VI)] are considered environmentally stable and biologically significant.^1–3^ Cr­(III) is recognized as an essential micronutrient that plays a vital role in glucose and lipid metabolism and is commonly used as a dietary supplement for humans and animals. In contrast, Cr­(VI) compounds are highly toxic and have been classified as human carcinogens due to their harmful effects following inhalation, ingestion, or dermal exposure.^4,5^ Given these toxicological concerns, several European regulatory bodies have emphasized the need to enhance standardized analytical methods for the detection and quantification of chromium species. These efforts aim to minimize environmental contamination and protect consumer health.^6^ As food naturally contains various metal ions, its consumption contributes to the daily intake of essential trace elements, including chromium.^7,8^ Consequently, the accurate determination of Cr­(VI) levels in aqueous samples is crucial for exposure assessment, dietary monitoring, and evaluation of the potential health risks. Accordingly, there is an urgent need for a fast screening method to determine or discriminate between different chromium species, Cr­(III) and Cr­(VI), in waters.

Currently, various modern methods have been reported to determine the concentration of Cr, such as AAS, ICP-MS, LC, LC-MS, and CE techniques.^9–12^ Regardless, they demand sophisticated instruments, multiple sample treatment steps, and trained operators, which make these methods time-consuming and costly for regular environmental surveillance.^6^ Alternative methods for easily and quickly sensing Cr species by colorimetry and electrochemistry have been developed.^10,13,14^ These approaches provide a high opportunity for integrating mobile detectors, multiplex detection, and high-throughput inspection. Among these, colorimetric sensing methods are favored primarily because they are easily observed through visual color changes. It can be widely employed for point-of-care utilization, especially in resource-poor settings. Despite that, the paramount objection to the above methods is their poor sensitivity to identifying low concentrations (μg L^–1^) of the analyte.

The nanosized materials are operative in the adsorption process due to their individual properties. Nanomaterials may be utilized as productive, eco-friendly, and cost-effective adsorbents for removing and extracting toxic species from water, such as heavy metals, azo dyes, etc.^15–18^ The adsorption features of nanomaterials can be improved by integrating them with other functionalizing agents. This combination is significant for the use of nanoparticles for preconcentration and separation processes. Until now, the adsorption of colored matter on white adsorbents has been used to evidence the existence of the substance in a mixture or as a preliminary to elution in the quantitative evaluation of the substance. When the measured quantity of acceptable white adsorbent is used, the density of color consisting of optimized terms is proportional to the dose of analyte in the sample. In this case, it is possible to estimate concentrations as low as 1.0 mg/L or less. Reflectance colorimetry is made visually of a color-growing agent on a solid material and is more straightforward to manufacture and handle. Reflectance colorimetry using an adsorbent (nanomaterial, resin, or fabric) is a trouble-free and selective method of colorimetric research based on naked-eye surveillance. To date, various sorbents, such as amine-functionalized mesoporous silica (AMS),^19^ cellulose-based solid amine sorbent,^20^ cationic waste cotton fabric,^3^ imidazolium-functionalized conjugated polymer,^21^ XAD-7HP,^22^ DPC immobilized alginate/pectin,^23^ and microfluidic paper,^24^ have been documented for Cr­(VI) detection. Up to now, the Cr­(VI) ions have been successfully detected by the above-mentioned approaches; no report has been published in the literature for the sensing of Cr­(VI) by FORS.

Heteropoly acids (HPAs) are a category of discrete anionic transition-metal oxides with beautiful properties.^25–27^ They have been considered as the probable green options for commonly used mineral acids owing to their tunable Brønsted or Lewis acidity, redox characteristics, thermal stability, etc.^28–32^ For example, cellulose acetate with a degree of substitution can be acquired by H_3_[PW_12_O_40_] catalyzed esterification of cellulose with acetic anhydride in CH_2_Cl_2_.^32^ Nonetheless, it is an even greater challenge in the wide utilization of HPAs due to the issues of their low BET surface areas (10 m^2^/g) and inconvenient recovery and separation. H_2_WO_4_ is used for the simple synthesis of W-oxides and other W-based nanomaterials. These W-based nanomaterials have implementations in sensors, energy storage, electrochemical devices, and photovoltaics. The chemistry of transition-metal (W, V, Mo, and so on) peroxy complexes has attracted significant interest over the past few years due to their importance in industrial, pharmaceutical, and biological processes.^33^ Therefore, peroxotungstic acid (PWA) compounds (or complexes) are currently of crucial interest. PWA is an essential precursor and can be employed in aqueous media to compose WO_3_. PWA is prepared *in situ* by reacting H_2_O_2_ with tungsten or tungstic acid. PWA compounds are well-known industrial catalysts and are probably just as substantial in biochemical systems. In coordination with a metal, peroxide gets activated, rendering the metal peroxides critical mediators in diverse biological and synthetic catalytic reactions. The PWA compounds all possess one to three peroxy groups and one or more mono- or bidentate organic or inorganic ligands. These ligands (anions, cations, and neutral molecules) can help to equalize the PWA complexes and can also affect the reactivity of the peroxy groups. PWA-based compounds are also of notable interest due to their collaboration in oxygen transfer reactions and many inorganic and organic reactions.^34^ Such reactions are considered eco-friendly and an option for many conventional oxidation reactions. Recently, several fascinating peroxy compounds of different early transition metals (W, V, Nb, Cr, Mo, Ti, Zr, etc.) have already been prepared and characterized.^34,35^ Previously, Das et al.^36^ prepared a polymer-bound PWA catalyst of the type [W­(O)_2_(O_2_)­(CN)_2_]–PAN [PAN = poly­(acrylonitrile)] (W-PAN) by reacting H_2_WO_4_ with 30% H_2_O_2_ and the macromolecular ligand, PAN, at pH 5.0. A polymer-linked peroxy molybdenum compound [MoO_2_(O_2_)­(CN)_2_]–PAN (Mo–PAN) was also synthesized by reacting H_2_MoO_4_with 30% H_2_O_2_.^34^ Furthermore, Kalita et al.^35^ developed a set of well-defined macro-complexes by incorporating vanadium peroxo complexes into water-soluble polymers (water-SP), viz, poly­(sodium acrylate) and poly­(sodium methacrylate). The noteworthy attributes of these polymeric (W, Mo, and V) compounds appear to be the first known instances of peroxo-metal compounds attached to water-SPs.^37^ Combining organic polymers with peroxo-metal complexes constitutes an operative area of current research,^38^ and the utility of WSPs as supporting materials in organic chemistry and biology is progressively being recognized in recent years.^39,40^ Polymer materials often lack various unsuitable features of monomeric species, such as liability, toxicity, volatility, and odor.^41,42^ Choice of the polymers is a crucial prerequisite to obtain a stable polymer–metal linkage. Nevertheless, with the huge advancement in the field of metal-containing polymers, there seems to be a dearth of knowledge about the synthesis and testing of peroxo-metal combinations attached to WSPs.

Inspired by the mentioned reports, we proposed the preparation of WSP-bound PWA nanoparticles (PDDA@PWA) via the reaction of H_2_WO_4_ with H_2_O_2_ and the cationic polyelectrolyte, PDDA, at ambient pH. These opaque white nanoparticles functioned as a selective solid-phase extraction (SPE) platform for trace Cr­(VI) ions. Following the SPE process, the FORS assay was directly performed on the Cr­(VI)-enriched PDDA@PWA nanoparticle (NP) surface using DPC as the chromogenic reagent. DPC was selected for its high sensitivity and specific colorimetric reaction, which forms a stable red-violet complex with Cr­(VI) exhibiting a reflectance peak at 518 nm. To validate the FORS assay results, the complexes (identified as Cr­(III)-DPCO) were carefully eluted from the nanoparticle surface and subsequently analyzed using FO–UV–Vis spectrophotometry.

## 2 Materials and Methods

### 2.1 Chemicals

Poly­(diallyl dimethylammonium) chloride, 20% in water with an average molecular weight between 200,000 and 350,000 Da, was provided by Sigma-Aldrich Chemical Co. CrCl_3_·6H_2_O and K_2_CrO_4_·6H_2_O salts were obtained from Merck. Cr­(III) and Cr­(VI) standard solutions were prepared in 1.0 mM HNO_3_· Other chemical reagents used Na_2_WO_4_·2H_2_O, H_2_O_2_ 30%, HCl 37%, HNO_3_ 69%, H_2_SO_4_ 96%, NaCl, ethanol, acetone, DPC, Na_2_HPO_4_·12H_2_O, NaH_2_PO_4_·2H_2_O, H_3_PO_4_, and CH_3_COOH (100%) were purchased from Sigma-Aldrich. Certified reference material (CRM) consisting of fortified lake water (TMDA-54.4) was obtained from the National Water Research Institute, Environment Canada (Burlington, ON, Canada). The low concentrations of Cr­(VI) and Cr­(III) standards were prepared by diluting the stock solutions on the day of the investigations. The DPC solution (5.0 × 10^–3^ M) was prepared fresh daily by dissolving 12.11 mg of DPC with 9 mL of ethanol, then completing the volume to 10 mL using 2.0 M H_2_SO_4_. The pH values of the samples were adjusted by using 0.1 M HCl and 0.1 M NaOH solutions. Distilled water was obtained from a laboratory water purification system and used in the research experiments. The Liebermann–Burchard reagent (LBR) was freshly prepared following the method described in the literature with slight modification.^43^ Briefly, 10.0 mL of glacial acetic acid was dispensed into an amber glass vial and equilibrated in an ice bath. After cooling for 5.0 min, 1.0 mL of concentrated H_2_SO_4_ was carefully added to the CH_3_COOH and mixed thoroughly to produce the LBR (CH_3_COOH:H_2_SO_4_, 10:1 v/v). Subsequently, 1.0 mL of the prepared LBR was combined with 1.0 mL of N, N-dimethylformamide (DMF) to yield a 1:1 (v/v) solution. This step was also performed under cooling (e.g., maintained in the ice bath) to prevent overheating or degradation of the solvent. The resulting mixture was employed as the eluent and designated as LBR@DMF. The accuracy of the proposed method was further evaluated by determining the total chromium content in the TMDA-54.4 CRM (certified Cr­(III) value: 438 ± 4.15 μg L^–1^). Before analysis, the Cr­(III) species present in the CRM were quantitatively oxidized to Cr­(VI) following established protocols described in the literature.^44,45^

### 2.2 Experimental Equipment

The solution pHs were checked using a pH meter (HANNA HI 221, Woonsocket, RI, USA). An Elmasonic E30H ultrasonic bath (Singen, Germany) was used for the agitation of samples. The morphology, structure, and composition of the synthesized PDDA@PWA NPs were characterized using scanning electron microscopy (Quanta 450 FEG-EDS field emission SEM), Fourier transform infrared spectroscopy (Agilent Cary 630 FTIR analyzer), X-ray diffraction and spectroscopy (Panalytical Empyrean XRD), X-ray photoelectron spectroscopy (Thermo Scientific K-α XPS), and high-resolution-transmission electron microscopy (TEM) (Hitachi HighTech HT7700 HR-TEM). Reflectance and ultraviolet–visible (UV–vis) measurements were performed using a commercially available miniature fiber-optic-based spectrometer (Ocean Optics Inc., HR4000CG-UV-NIR), which utilizes a small tungsten halogen lamp (Ocean Optics Inc.) as the light source and a charge-coupled device (CCD) based detector for reflectance measurements. Furthermore, the reflectance measurement setup is used to measure the reflection from the sample. A schematic illustration of the angle-dependent reflectance spectrum measurement setup is described in Figure 1. The sampling port was made by cutting it from a 96-well microplate (diameter: 6.94 mm, depth:10.85 mm, volume: 400 μL). For optical isolation, the sensing layer and the detector were kept in a black box to minimize any interference from ambient light. As can be seen in the picture of the reflection setup, the fiber optic reflection probe is placed close to the sample. The optimal distance between the sample and probe is approximately 4–5 mm. It consists of a broadband halogen-tungsten light source (HL-2000-HP-FHSA, Ocean Optics Inc., FL), a fiber-coupled cuvette holder with two collimating lenses (CUV-ATT-DA, Avantes Inc., Netherlands), and a USB portable spectrometer (USB4000 FL, Ocean Optics Inc.).

**Figure 1:**
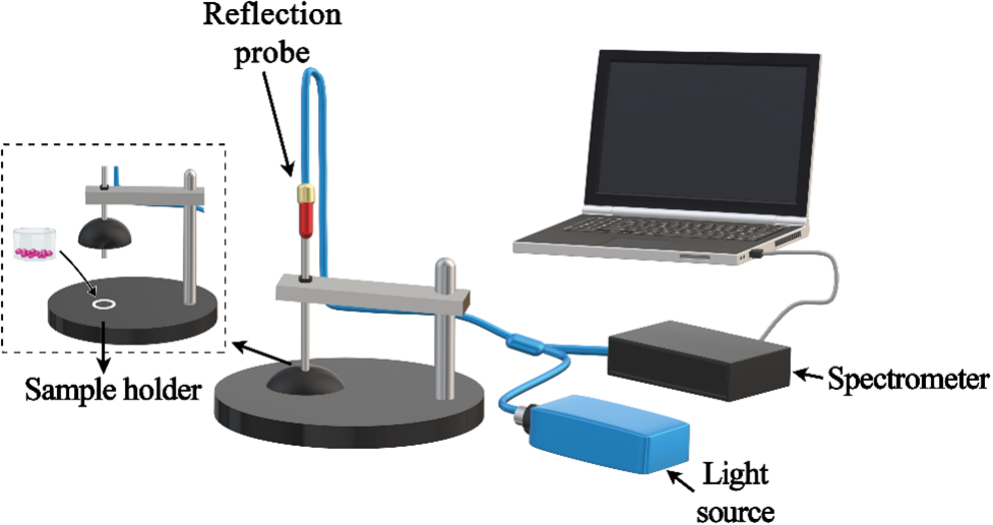
Schematic representation of the FORS setup used for Cr­(VI) measurement. (Figure was created by authors with Microsoft Paint 3D).

### 2.3 Synthesis of PWA

A peroxo polytungstate­(VI) anion decorated WSP was obtained by reacting H_2_WO_4_ with H_2_O_2_ and the cationic polyelectrolyte, PDDA, at mildly acidic pH.^46^ H_2_WO_4_ is known for its acidic properties, exhibiting a pH lower than 7.0 in solution, and it can act as a weak acid. H_2_WO_4_ was synthesized according to a previously reported protocol, with slight modifications.^47^ Briefly, 4.0 g of Na_2_WO_4_ ·2H_2_O was thoroughly dissolved in 50 mL of distilled water. Then. Ten mL of HNO_3_ (65% w/w) was added dropwise with vigorous stirring at ∼50 °C until the pH of the solution was *<*1. A pale-yellow precipitate (H_2_WO_4_) was obtained after being continuously stirred for 8 h. The solid particles were then filtered and washed with distilled water and alcohol several times to remove ions that stayed in the solids and dried at 60 °C. Following the above procedures, a higher yield (∼2.9 g) and high purity of H_2_WO_4_ were obtained. After that, the synthesis of PWA was carried out. The synthesis conditions were previously reported by Gong et al.^46^ The synthesis of PWA was based on a well-known procedure with some minor modifications.^46^ Briefly, 1.0 g of H_2_WO_4_ dissolved in 50 mL of 3.0 M H_2_O_2_ (a liquid–solid ratio of 50:1). The pH of the solution was carefully adjusted to 4.0 with acetic acid (If needed). The mixture was stirred at room temperature for 1.0 h. The PWA sol was formed via dissolving H_2_WO_4_ precipitate in 50 mL of H_2_O_2_ (3.0 M H_2_O_2_). The synthesis reaction can be expressed as^46^

1 $${\mathrm{Na}}_{2}{\mathrm{WO}}_{4}+2{\mathrm{HNO}}_{3}\to H_{2}{\mathrm{WO}}_{4}↓+2{\mathrm{NaNO}}_{3}$$

2 $$2H_{2}{\mathrm{WO}}_{4}+4H_{2}O_{2}\to H_{2}W_{2}O_{11}+5H_{2}O$$

### 2.4 Preparation of PDDA@PWA

The PWA complex is reported to be W_2_O_11_^2–^, [W_2_O_3_(O_2_)_4_]^2–^ or ([(O)­W­(O_2_)_2_(O)­(O_2_)_2_W­(O)]^2–^), where (O_2_) denotes a peroxo ligand.^48,49^ In this system, H_2_O_2_ acts as both an oxidizing agent and a complexing agent.^46^ PWA has very low p*K*_a_ values, often comparable to or stronger than that of sulfuric acid. The PWA dissolves readily in water because of its polar nature and large hydrated ions. When dissolved, they produce H^+^ ions effectively, giving rise to strongly acidic solutions. The PDDA@PWA NPs were prepared following a previously reported method^50^ with slight modifications. Subsequently, 1.0 g of PDDA (20% w/v) (PDDA/H_2_WO_4_ ratio of 1:1) was added and stirred at 500 rpm at room temperature for 1.0 h. During the reaction, the chloride anion in PDDA acts only as a counterion and does not participate in the formation of the nanoparticle. The actual nanocomposite is formed by the interaction of the cationic PDDA polymer with the anionic peroxopolytungstic acid species, with chloride being displaced and not incorporated into the final material. Finally, the white precipitate was centrifuged, repeatedly washed with distilled water, and then washed with ethanol and dried at 40 °C for 3.0 h. The obtained masses were carefully crushed in a mortar and subsequently heated at 100 °C for 4 h. As a result, the white powders of PDDA@PWA NPs were obtained. To show the reproducibility of PDDA@PWA NPs, the synthesis process was repeated three times. The reaction conditions are as follows: an H_2_O_2_ concentration of 3.0 mol/L, pH of 4.0, a liquid–solid ratio of 50:1, a reaction temperature of 25 °C, and a reaction time of 1.0 h (PDDA/H_2_WO_4_ ratio of 1:1, w/w).

### 2.5 Recommended FORS Procedure

The adsorption mechanism relies on electrostatic attraction between the positively charged surface of the PDDA@PWA adsorbent and the negatively charged Cr­(VI) species (HCrO_4_^–^). For the SPE procedure, an aliquot of Cr­(VI) solution (1.0 mL; concentration range: 0.2–6.0 μM) was combined with 9.0 mL of HCl solution (adjusted to pH 4.0) in a 10 mL centrifuge tube. Subsequently, 10 mg of PDDA@PWA adsorbent was introduced to the mixture. The resulting suspension was agitated for 20 min to ensure thermodynamic equilibrium between the analyte and the adsorbent surface. Phase separation was achieved by centrifugation at 5000 rpm for 5 min. The supernatant was carefully decanted, and the Cr­(VI)-loaded PDDA@PWA nanoparticles were retained. To eliminate unbound ions, the solid phase was washed twice with 1.0 mL of distilled water. Following extraction, the Cr­(VI)-loaded nanoparticles were redispersed in 70 μL of ethanol, vortexed for 1.0 min, and transferred to a sampling cell. The slurry was dried at 45 °C for 10 min under ambient conditions to form a uniform layer. For the colorimetric reaction, 20 μL of a 5.0 × 10^–3^ M DPC solution was applied directly to the dried PDDA@PWA layer. In a dark, enclosed environment, the reaction was incubated for 10 min. This was done to encourage solvent evaporation and aid in the formation of the characteristic Cr­(III)–DPCO complex. Both DPC and its oxidized product (DPCO) are photosensitive and are susceptible to degradation or fading when exposed to strong ambient light. Reflectance spectra were subsequently acquired using the FORS system with the sampling cell positioned in the test port. The reflectance signal (*R*%) was measured at each wavelength, ensuring that the stability of the colored complex (Cr­(III)-DPCO) was maintained throughout the analysis. The experimental setup and FORS procedure are schematically illustrated in Figure 2.

**Figure 2:**
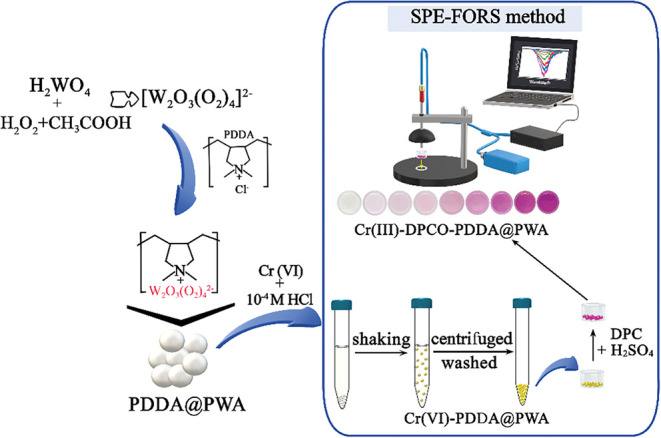
Schematic illustration of the integrated analytical workflow for Cr­(VI) determination. (Figure was created by authors with Microsoft Paint 3D).

## 3 Results and Discussion

### 3.1 Characterization of the Adsorbent

#### 3.1.1 XRD Analysis

As shown in Figure 3A (a), the sharp diffraction peaks located at the 2θ values of 16.3, 25.6, 32.6°, and 35.8°, and the other well-resolved, fragmented, and less intense peaks were assigned to the development of the orthorhombic crystalline structure of WO_3_·H_2_O (H_2_WO_4_). The high intensities of the peaks verify the good crystalline character of the synthesized H_2_WO_4_. The specific XRD peaks of H_2_WO_4_, (020), (111), and (131), are in good concurrence with the standard card of orthorhombic architecture (JCPDS 43–0679). The H_2_WO_4_ is relatively clean and entirely in agreement with WO_3_H_2_O (JCPDS card no.35–1001).^51–53^ After the oxidation of H_2_WO_4_, the recorded patterns are illustrated in Figure 3A (b). The XRD peaks at around (2θ) 12.5°, 22.9°, 23.3°, 24.1°, 26.5°, 28.1°, 32.8°, 33.2°, 33.9°, 41.5°, 49.83°, and 50.44° can be assigned to the (011), (002), (020), (200), (120), (112), (022), (202), (220), (222), (232), and (114) crystalline planes of the monoclinic phase of peroxotungstic acid (PWA) [W_2_O_3_(O_2_)_4_(H_2_O)_2_]^2–^. The results are in good agreement with the standard JCPDS card No. 83–0950. Notably, the typical peaks for pure H_2_WO_4_ and PWA are observed at ∼12.6° and ∼16.5°, respectively. Thus, the broadened peak located at ∼12.6° confirms the synthesis of PWA.^46,54^ In the XRD of PDDA@PWA (Figure 3A­(d)), the strong peaks at 2θ = 10.50°, 16.27, 21.45°, 27.48°, 31.79°, and 34.10° are assigned to the peroxo-structure of PDDA@PWA [JCPDS File 50–0657], respectively. Research results show that the structure of H_2_WO_4_ is completely or partially destroyed as an outcome of the interaction with H_2_O_2_ to form peroxide-containing H_2_W_2_O_11_ species. It can also be seen that PDDA@PWA has a lower peak intensity. The results can be interpreted as evidence that H_2_W_2_O_11_ species are finely and molecularly dispersed on the PDDA surface through chemical interactions, resulting in strong binding of polyanions on the cationic PDDA matrix as charge-balancing components.

**Figure 3:**
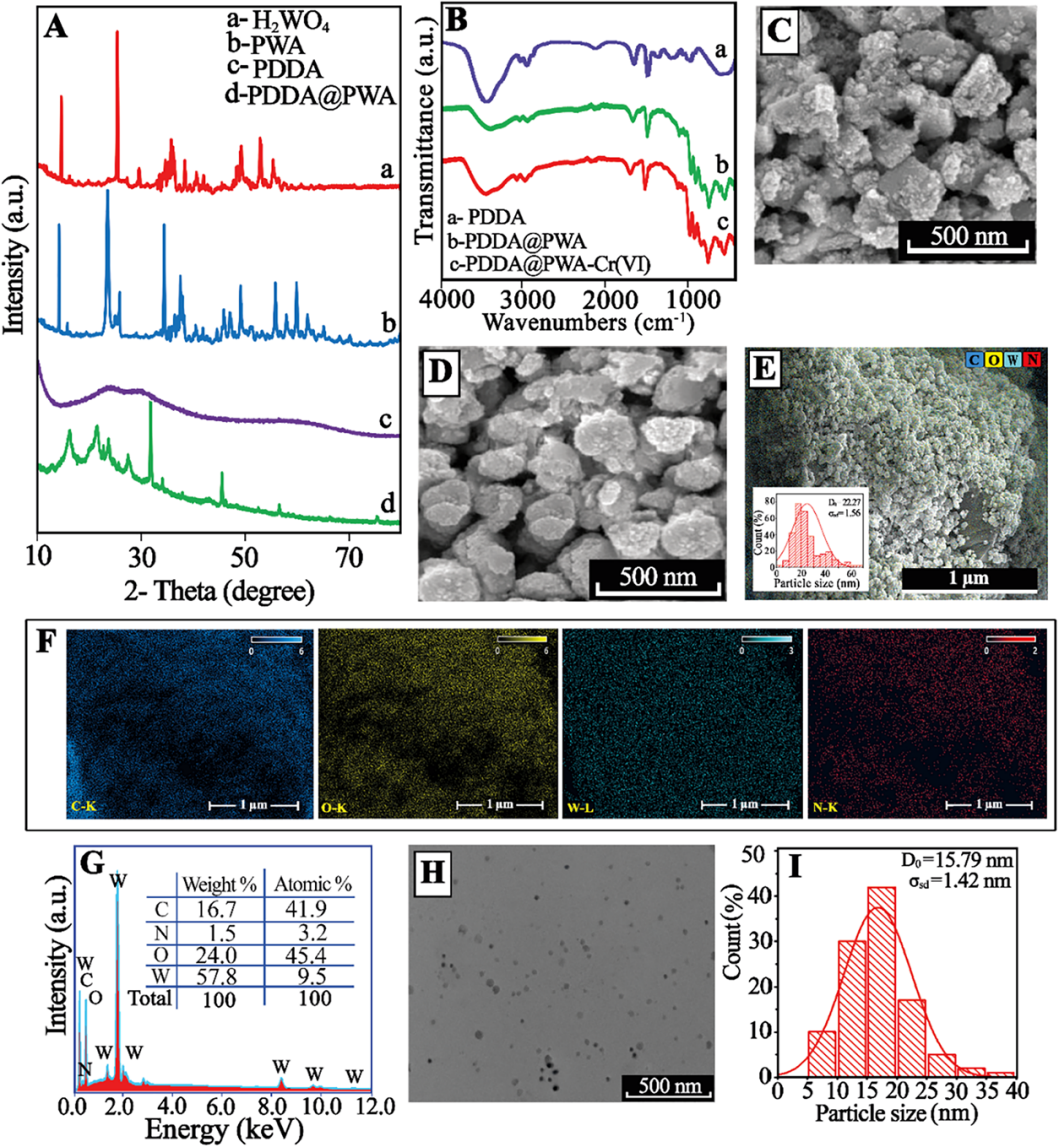
(A) XRD pattern of (a) H_2_WO_4_, (b) PWA, (c) PDDA, and (d) PDDA@PWA. (B) FTIR spectra of the (a) PDDA, (b) PDDA@PWA, and (c) PDDA@PWA-Cr­(VI). SEM images of (C) pure H_2_WO_4_, (D) PWA, and (E) PDDA@PWA (Inset: A particle size distribution histogram determined from SEM). (F) Elemental mapping analysis of the PDDA@PWA, (G) EDX spectra of the PDDA@PWA, (H) TEM images of the PDDA@PWA, (I) A particle size distribution histogram determined from TEM.

#### 3.1.2 FTIR Analysis

FTIR spectroscopy was employed to characterize the PDDA compound and elucidate the functional groups present in the cationic polymer. Figure 3B exhibits the FTIR spectra for PDDA (a) and PDDA loaded with PWA (b). The cationic polymer exhibits a typical peak at 3434 cm^–1^, corresponding to N–R_4_ groups. Furthermore, the peaks at 3018 and 2942 cm^–1^ correspond to the C–H binding stretching vibrations of −CH, −CH_2_, and −CH_3_ groups. The band at 1475 cm^–1^ is related to the methylamine group (−N^+^–(CH_3_)_3_), and the peak at 1341 cm^–1^ indicates the C–H bond.^55^ In the FTIR spectra of PDDA@PWA NPs, the absorption peak corresponding to the vibration of the terminal (−N^+^–(CH_3_)_3_) group shifts from 1475 cm^–1^ to 1471 cm^–1^. On the other hand, the broad peak at 839 cm^–1^ may be attributed to a vibration mode due to the W–O–W bridge. It confirms the presence of a peroxogroup (W–O–O–W) in the W–O networks in PWA. The above findings indicate that the unification of PWA into the PDDA matrix introduced a reaction between PWA and PDDA molecules, but at a lower FTIR intensity.^56^

Finally, Cr­(VI) adsorption onto PDDA@PWA NPs was also studied by using FTIR analysis. The change in the FTIR spectra of PDDA@PWA-Cr­(VI) was monitored as shown in Figure 3B (curve *c*). A new strong peak ascribed to the Cr–O stretching vibrations was observed at 940 cm^–1^ after Cr­(VI) adsorption. These findings confirm that Cr­(VI) retention occurs predominantly through electrostatic interactions with protonated quaternary amino groups.

#### 3.1.3 SEM and TEM Analysis of PDDA@PWA

The SEM image analysis was performed to understand the surface morphology of PDDA@PWA composites. Figure 3E illustrates the lower resolution and highly resolved SEM images of the PDDA@PWA sample, which exhibited an irregular morphology and a cracked surface area. This irregular morphology suggests that the composite may have complex structural characteristics that could influence its performance in various applications. Elemental mapping and EDX spectra (Figure 3F,3G) confirm the stoichiometric composition within the prepared PDDA@PWA composite. The appropriate metal ion peaks, such as oxygen (O), tungsten (W), and nitrogen (N), observed in the PDDA@PWA spectra confirm the formation of the PDDA@PWA material. To further observe the microstructure of PDDA@PWA, the TEM analysis was carried out as shown in Figure 3H. Some of the PWA particles in PDDA@PWA material are larger blocks (Figure 3H); however, most of them become smaller particles with around ∼15.79 nm (mean) diameter, and they are homogeneously anchored onto the PDDA layers. The NP sizes were estimated utilizing a nanomeasurement system, and the statistical data were plotted in Figure 3H.^57^ The particle size histogram (Figure 3H) shows a size range from ∼5 to ∼38 nm. According to the SEM images (Figure 3E), the PDDA@PWA NPs show a size range from ∼5 to ∼66 nm. As a result, the TEM shows the size of nanoparticles is around 5 nm or less, while the SEM shows the size is above ∼22.27 nm. This can be explained as follows: TEM achieves subnanometer resolution for internal structures, while the SEM image provides superior surface topography with a resolution of 1–5 nm. TEM can give indispensable knowledge regarding the inner structure of target samples, such as crystal structure, stress state information, and morphology at the atomic scale. In contrast, an SEM image offers valuable insight into the sample’s 3D surface and composition. Briefly, if you need to view a relatively large area and only require surface features, SEM analysis is unique. If you require inner inspection of small samples at near-atomic resolution, TEM analysis will be mandatory. Furthermore, for an SEM analysis of powdered materials, some powders are usually utilized. In the case of a TEM analysis, the analyzed material is dispersed in a suitable solvent by a high-power ultrasonic instrument to spread the sample well. Then, the dispersion was placed onto a TEM grid, and the sample was fully dried in air to reach a thinly sliced sample. Therefore, the images acquired from TEM have higher resolution, and the agglomeration (or touching) of nanoparticles could be distinguished clearly with TEM, while in an SEM image, the agglomerated (or touched) nanoparticles are normally monitored with larger sizes. TEM images provide direct monitoring of the shape and size of individual NPs on a substrate, even with the presence of large aggregates, leading to accurate estimations of particle size. SEM is a highly realistic imaging tool; nevertheless, it is not precise for measuring the size of the NPs.^58–61^ Lastly, Figure 3E shows SEM images of PDDA@PWA, illustrating their morphological properties. The structure of PDDA@PWA was noted to have a spherical form and an uneven distribution of particles with sizes ranging from ∼5 to ∼66 nm (mean: ∼22 nm). The estimated histogram determined from SEM is shown in Figure 3E (inset). Furthermore, Figure 3H shows a TEM image of PDDA@PWA NPs. It exhibits that the PDDA@PWA NPs were well-distributed in form and size, ranging from ∼5 to ∼38 nm. Figure 3I shows the PDDA@PWA size distribution histogram determined from TEM. Most of the PDDA@PWA NPs were seen to be spherical.

#### 3.1.4 XPS Analysis

XPS analysis is carried out to explore the chemical state of the elements present in PDDA@PWA through the binding energy (BE). The survey spectra of PDDA@PWA (blue curve) and PDDA@PWA-Cr­(VI) (red curve) are illustrated in Figure 4A. The survey spectra of PDDA@PWA (blue curve) exhibit the presence of C 1s (286.08 eV), N 1s (402.08 eV), W 4f (39.08 eV), and O 1s (533.08 eV) core levels with no verification of dirtiness. Figure 4B displays the O 1s spectra; one at a BE of 530.8 eV is defined as the oxygen in W–O, and the second, rather small peak at 531.98 eV (Figure 4B) could indicate water molecules retained on the nanomaterial surface. Figure 4C displays the N 1s spectra of the PDDA@PWA sample. After the fitting procedure, two crucial peaks appear. The first one is broad and located above 402.18 eV and is specific for C–N and C=N bonds with sp^2^ hybridization. The second peak is also a broad one, but it is less intense and located above 399.18 eV, and is distinctive for C–N bonds with sp^3^-hybridization. The spectra of C 1s in Figure 4D were simplified into two typical peaks with BEs of 284.81 and 288.15 eV, respectively. The former is specified as the C–C bonds, and the latter is attributed to the three-coordinated C atoms (N–C=N–C) in the N-containing framework. Nevertheless, Figure 4E displays the W 4f spectra with two specific peaks located at BEs of 35.68 and 37.88 eV. The W 4f_7/2_ (at 35.68 eV) is attributed to PWA architecture. The XPS findings confirm the presence of PDDA, PWA, and N in the architecture in conjunction with the results of the FTIR spectra.

**Figure 4:**
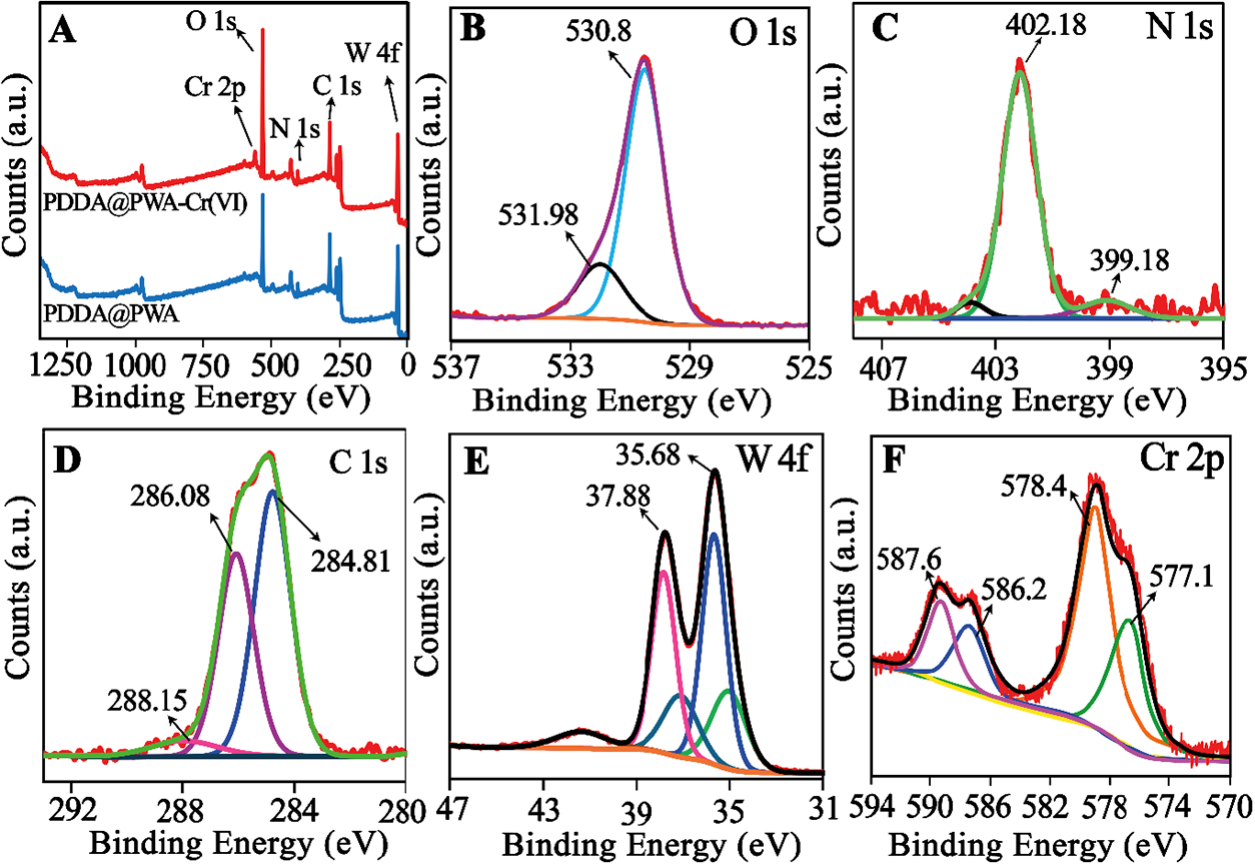
(A) XPS survey spectra of PDDA@PWA and PDDA@PWA-Cr­(VI). High-resolution XPS spectra of (B) O 1s, (C) N 1s, (D) C 1s, (E) W 4f, and (F) Cr 2p in PDDA@PWA.

Besides, XPS analysis was employed to provide the valency state of Cr­(VI) loaded onto the adsorbent (Figure 4A, red curve). Five peaks of Cr, W, O, C, and N can be seen in low-resolution XPS spectra of the Cr­(VI)-loaded adsorbent. The survey results confirmed Cr­(VI) species coexist in the composite. Figure 4F shows the deconvolution of the high-resolution Cr 2p spectra. As shown in Figure 4F, the BEs of the Cr 2p_3/2_ and Cr 2p_1/2_ peaks are located at 577.1 and 586.2 eV, belonging to 2p_3/2_ and 2p_1**/2**_ of Cr­(III).^62,63^ Meanwhile, the BEs of Cr 2p_3/2_ and Cr 2p_1/2_ peaks at 578.4 and 587.6 eV correspond to the Cr­(VI) states.^64^ Seen from Figure 4F, Cr 2p peaks can be curve-fitted with four components at binding energies of 587.6 eV, 586.2 eV, 578.4, and 577.1 eV; the peak components at 577.1 and 578.4 eV correspond to Cr 2p_3/2_ orbitals, while those at 586.2 and 587.6 eV correspond to Cr 2p_1/2_ orbitals. The peaks at binding energies of 578.4 and 587.6 eV can be regarded as the peaks of Cr­(VI), while the peaks at 577.1 and 586.2 eV can be attributed to Cr­(III). The result implies that some of the Cr­(VI) species are also reduced to Cr­(III) after the adsorption process. A similar reduction during the adsorption process was previously observed by Li et al.^21^ and attributed to the mildly reducing nature of electron-rich quaternary amine groups. This implies that both Cr­(VI) and Cr­(III) exist on PDDA@PWA. Cr­(VI) removal by PDDA-modified materials (such as PDDA@PWA) involves a reduction-adsorption mechanism where Cr­(VI) is reduced to Cr­(III). The positively charged PDDA coating increases the electrostatic attraction of anionic Cr­(VI), after which reduction occurs, and ultimately, some Cr­(III) is formed on the surface of the material.

#### 3.1.5 Measurement of ζ-Potential (ZP)

ZP is another crucial characteristic of nanoformulations. As testified by ZP measurements, (a) PWA NPs were negatively charged with a ZP of −42.5 ± 2.01 mV, while (b) PDDA@PWA NPs became positively charged with a ZP of 58.1 ± 1.73 mV, as shown in Figure 5. PDDA is a WSP (quaternary ammonium) and a strong polyelectrolyte. W_2_O_11_^2–^ anions possess a discrete ionic texture, including heteropolyanions (W_2_O_11_^2–^) and counter cations. Therefore, these W_2_O_11_^2–^ anions are easily self-assembled onto the PDDA surface by electrostatic interaction between the positively charged PDDA and negatively charged (W_2_O_11_^2–^) ions. During the preparation of PDDA@PWA NPs, it was observed that pale-yellow solids formed when the PDDA and PWA solutions were combined, indicating a strong interaction between the PDDA and PWA molecules. The ZP is the electrical potential that exists at the shear plane of suspended NPs in solution. As shown in Figure 5, the ZP of the PWA (ZP: −42.5 ± 2.01 mV) changes significantly after mixing with PDDA solutions (ZP: 58.1 ± 1.73 mV). The positive ZP for PDDA@PWA NPs is apparently an indication of the functionalization of PWA by the positively charged PDDA; meanwhile, the negative ZP for PWA shows the successful assembly of PWA anions on the PDDA polymer by electrostatic force. To further elucidate the pH dependence of Cr­(VI) adsorption on PDDA@PWA, the surface charge of the adsorbent was characterized by measuring its ZP across a pH range from 1.0 to 7.0. As depicted in Figure 5C, the net charge of PDDA@PWA displayed a trend of initially increasing from +55.3 to +58.1 mV and then decreasing to +35.3 mV as the pH value increased. This trend correlates with the observed changes in Cr­(VI) adsorption capacity and detection efficiency. Notably, PDDA@PWA maintains a strong positive surface charge across a wide pH range (1.0–7.0), which significantly enhances its affinity for negatively charged Cr­(VI) ions through electrostatic attraction.

**Figure 5:**
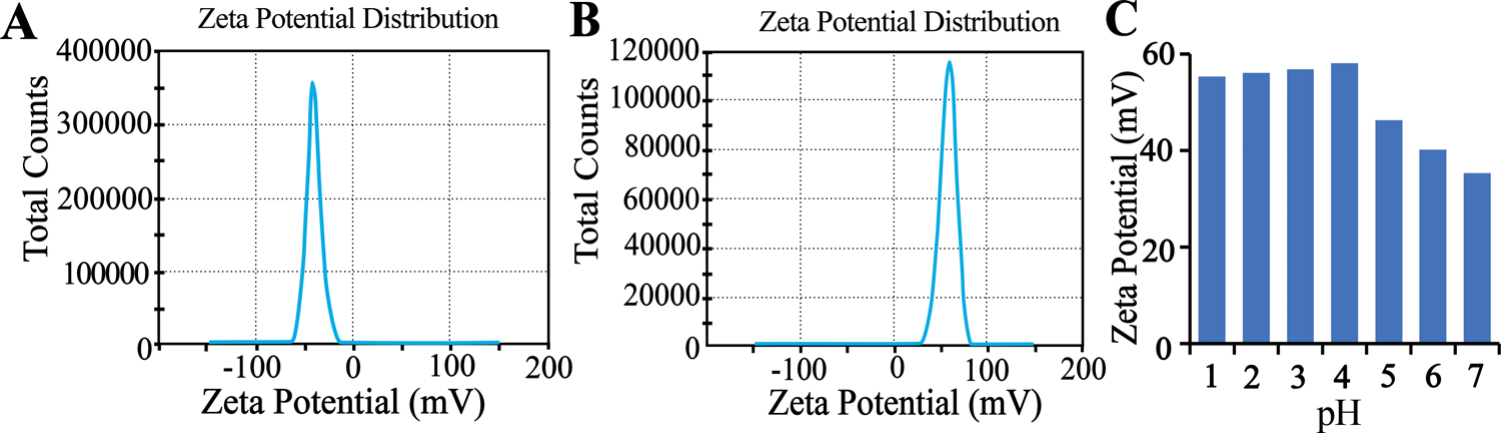
ζ-Potential of (A) PWA and (B) PDDA@PWA suspension. (C) ζ-Potential of PDDA@PWA at variable pH.

### 3.2 Optimization of Reflectance Assay Parameters

To maximize the SPE efficiency and achieve optimal detection sensitivity, systematic optimization of parameters influencing the extraction process is crucial. Each experimental condition was subjected to triplicate measurements. The adsorbent material (PDDA@PWA) was stored in a desiccator to prevent moisture adsorption. The adsorbent (15 mg) was immersed in 400 μL of LBR@DMF for 10 min and washed twice with distilled water before use. The adsorption behavior was investigated by using batch experiments at room temperature. For all SPE studies, 1.0 mL of 2.0 × 10^–5^ M Cr­(VI) was used; it was further diluted with 10^–4^ M HCl (final concentration is 2.0 × 10^–6^ M). After the adsorption process, the reflectance (*R*%) intensity of the colored adsorbents was recorded in the wavelength range of 350–800 nm. The measurements were taken relative to a blank SPE sample to assess the change in optical properties after Cr­(VI) adsorption.

#### 3.2.1 Effect of the Dosage of the Adsorbent

The nanoparticles act as solid supports to selectively extract and concentrate trace Cr­(VI) ions from the sample solution. Consequently, the amount of adsorbent significantly affects the extraction efficiency. Different dosages of the adsorbent can change the quantity of adsorbed ions and, subsequently, the reflectance intensity. The effect of this crucial factor was investigated within the range of 5.0–50 mg. As the PDDA@PWA content increased from 5.0 to 15 mg, the reflectance intensity increased progressively, reaching a peak at 15 mg, indicating that this loading provides optimal optical performance. The data obtained demonstrated considerable *R*% rates for Cr­(VI) in the range of 5.0–8.0% with a relative standard deviation less than 5%. The optimal adsorbent mass (15 mg per 10 mL of sample) ensures a balance between sufficient adsorption and a strong *R*% signal. When the amount of adsorbent is increased, the total surface area and the number of active binding sites available for adsorption also increase. This allows more Cr­(VI) ions from the aqueous solution to interact with and attach to the adsorbent surface, thus improving overall reflectance intensity. Briefly, an appropriate amount of the adsorbent can quantitatively extract Cr­(VI) from the solution. When the adsorbent dosage is below the optimum value, not all Cr­(VI) ions in the solution can be captured by the adsorbent because the surface area and active sites are limited. The *R*% intensity of Cr­(VI) on PDDA@PWA as a function of PDDA@PWA dosage is shown in Figure 6A. However, at higher doses, such as 15 mg, the reflectance intensity decreased monotonically. Increasing the adsorbent dose provides more available adsorption sites, which leads to a lower surface analyte concentration per unit mass of the adsorbent. As a result, the analyte’s reflectance intensity on the adsorbent surface decreases. In this study, 15 mg of adsorbent was used for the subsequent FORS experiments. Furthermore, the PDDA@PWA showed superior Cr­(VI) capture due to the relatively high positive ζ-potential (ZP: 58.1 ± 1.73 mV) and loading of PWA (ZP: −42.5 ± 2.01 mV). PDDA^+^ modifies the surface charge properties of PWA, thereby creating a favorable adsorbent for the selective adsorption of Cr­(VI) even in the presence of a certain amount of Cr­(III). Briefly, PDDA@PWA shows the preferential adsorption of Cr­(VI) over Cr­(III) due to charge-based interactions. The findings demonstrate the high potential of PDDA@PWA NPs for analytical and environmental applications, particularly in the monitoring and removal of Cr­(VI) from contaminated systems.

**Figure 6:**
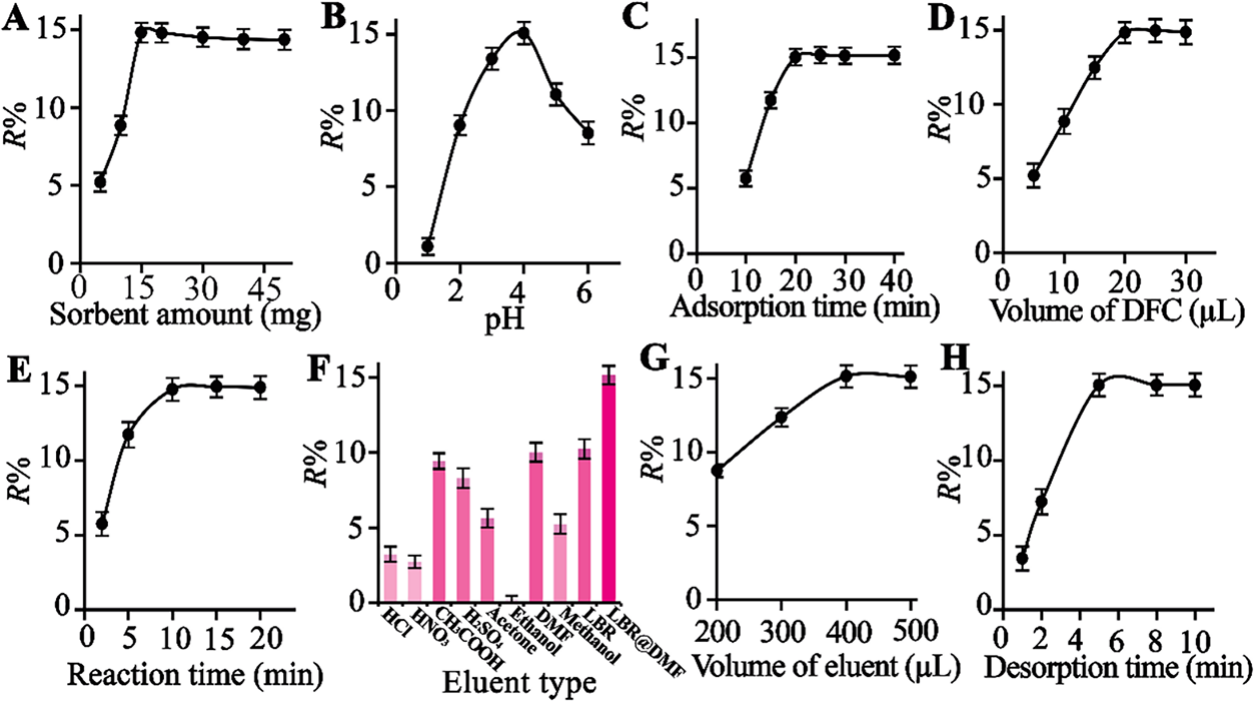
Effects of different parameters on the reflectance intensity of 2.0 μM Cr­(VI): (A) PDDA@PWA amount, (B) pH, (C) adsorption time, (D) volume of DPC, (E) reaction time, (F) eluent type, (G) volume of eluent, and (H) reusability. The sample volume is 10 mL.

#### 3.2.2 Optimization of pH

The pH of the solution plays a crucial role in determining the retention efficiency of Cr­(VI) ions onto PDDA@PWA. This is because the pH influences both the surface charge of the PDDA@PWA adsorbent and the predominant chemical species of chromium existing in solution. At lower pH values, the surface of PDDA@PWA tends to be more positively charged, which enhances the electrostatic attraction toward negatively charged Cr­(VI) species. Cr­(VI) ions can exist in several stable forms, such as Cr_2_O_7_^2–^, HCr_2_O_7_^–^, HCrO_4_^–^, and CrO_4_^2–^, and the relative abundance of a particular complex depends on the concentration of the chromium ion and the pH of the solution. Therefore, the effect of the test solution’s pH on the analyte’s reflectance was investigated. In this context, the reflectance intensity of Cr­(VI) was evaluated in HCl solution over a pH range of 1.0–6.0, as shown in Figure 6B. It is worth noting that the prepared novel adsorbent exhibited instability at pH values above 7.0. The adsorbent may lose stability or break down under weakly basic conditions. The reflectance intensity exhibited a maximum at pH 4.0, suggesting optimal optical properties under mildly acidic conditions. Beyond this point, reflectance intensity decreased, likely due to alterations in surface chemistry or molecular state. At pH ≈ 4, HCrO_4_^–^ predominates as the Cr­(VI) species. In the PDDA^+^/PWA multilayer system, some PDDA^+^ units bind directly to PWA^–^, losing their Cl^–^ counterions, while others remain as PDDA/Cl. The latter regions provide exchangeable chloride sites, allowing substitution by HCrO_4_^–^ via ion exchange. Thus, the adsorption of Cr­(VI) is governed by electrostatic and ion-exchange interactions between HCrO_4_^–^ and the PDDA^+^ matrix. At this pH, the negatively charged hydrogen chromate ions (HCrO_4_^–^) are electrostatically attracted to the positively charged quaternary ammonium groups on the PDDA@PWA surface, and the reflectance intensity of Cr­(VI) was highest, with a *R*% of 15% (RSD = 6.25%, *n* = 3). Conversely, low reflectance values are observed in the pH range from 1.0 to 3.0. The reduction in adsorption performance observed at pH values below 4.0 can be ascribed to the competitive occupation of adsorption sites by Cl^–^ ions added for pH regulation, corroborating the conclusions of Yu et al.^65^ Based on the results, the reflectance efficiency of Cr­(VI) decreases markedly at pH values above 4.0, with *R*% ranging from approximately 11.0% (pH 5.0) to 8.5% (pH 6.0) (RSD = 5.35% and 4.15%, *n* = 3) The findings emphasize that precise pH control is crucial in practical applications, as small deviations can markedly affect the efficiency of Cr­(VI) adsorption. Additionally, Cr­(III) ions were not adsorbed at all within the pH range of 1.0–4.0, and the reflectance intensity remained equal to that of the blank. This phenomenon can be ascribed to protonation of the PDDA@PWA surface, resulting in the generation of a net positive charge. Under these test conditions, the Cr­(III) ions, being positively charged, are repelled by the positively charged surface, which consequently limits their adsorption. Accordingly, the following FORS tests were conducted at a pH of 4.0 using a HCl solution.

#### 3.2.3 Effect of Adsorption Time

Adsorption time significantly influences the reflectance value of Cr­(VI) due to its effect on the interaction between the analyte and the adsorbent. Increasing contact time enhances the surface contact area and promotes the adsorption of Cr­(VI) ions, resulting in a higher reflectance intensity. Therefore, the adsorption shaking times varied from 1 to 30 min (5 min intervals). According to the test results, the adsorption of Cr­(VI) occurred rapidly within the first few minutes, with reflectance intensity rising from 5.0% to 15.02% (RSD = 5.14% and 5.85% *n* = 3). This rapid adsorption was attributed to the presence of PDDA^+^, which offers abundant ion-exchange sites, facilitating efficient interaction between HCrO_4_^–^ ions and PDDA^+^ functional groups.^66^ After about 20 min, the increase in reflectance intensity slowed, and the system approached equilibrium by 40 min, reaching a maximum reflectance intensity of 15.09% (RSD = 5.85%, *n* = 3). As shown in Figure 6C, the reflectance plateau at 20 min indicates that this is the optimal contact time for SPE of Cr­(VI) ions.

#### 3.2.4 Effect of DPC Concentration

Detection of hexavalent chromium [Cr­(VI)] on nanoparticle surfaces can be achieved using DPC, which reacts with Cr­(VI) to form a distinct red-violet colored complex. The coloration results from an intramolecular charge transfer process, rather than a d–d transition, since the d-orbitals of Cr­(VI) are not fully occupied.^67^ At an acidic pH of 1.0, the reaction between Cr­(VI) and DPC proceeds optimally (as supported by ASTM D1687–17, 2017).^68^ The cationic polymer PDDA^+^ serves as an Cr­(VI) adsorbent. The interaction strength between Cr­(VI) species and the PDDA surface depends on the steric bulk of substituents on the N atom—smaller substituents (such as methyl groups) enhance interaction and facilitate complex formation at the solid surface.^69^ The concentration of DPC directly affects the extent of color formation and thus the reflectance intensity measured by FORS. To optimize reagent use, varying volumes of 5.0 × 10^–3^ M DPC solution (5, 10, 15, 20, 25, and 30 μL) were tested. According to the test results, the reflectance intensity shows an increasing trend for the DPC volumes from 5.0 to 20 μL (Figure 6D). The reagent volume of 20 μL achieved full wetting of the adsorbent surface and complete color development (white-to-red-violet transition). Reflectance intensity reached its maximum. At higher concentrations, reflectance intensity plateaued; the excess DPC did not further enhance color formation, and quantitative measurements became less reliable due to saturation effects. Additionally, high ligand concentrations may alter local chemical conditions (e.g., effective pH), impairing the Cr­(VI)–DPC reaction efficiency. Volumes lower than 20 μL, incomplete color development (white-to-pale pink), indicating insufficient DPC to react with surface-adsorbed Cr­(VI) fully. The optimal DPC volume for quantitative Cr­(VI) detection using the solid-phase-based-FORS method was determined to be 20 μL of 5.0 × 10^–3^ M DPC solution. This condition ensures complete complexation, reduces reagent consumption, and provides stable and reproducible reflectance peak readings for quantification of Cr­(VI).

#### 3.2.5 Reaction Time for Color Development

The effect of the interaction time on color development was investigated. Following the SPE process, an optimized DPC reagent (20 μL of 5.0 × 10^–3^ M) was added dropwise onto the solid surface of Cr­(VI)–PDDA@PWA. The reflectance intensities of the resulting-colored complex were recorded at predetermined time intervals ranging from 2 to 20 min to determine the optimal reaction time for complete color development (Figure 6E). The solid samples were stored in a dark, preferably cool environment to prevent external light exposure. During this process, the DPC is oxidized to diphenyl carbazone (DPCO), and Cr­(VI) is reduced to Cr­(III), which then chelates to form a colored complex. Consequently, Cr­(III)–DPCO complexes are generated on the PDDA@PWA nano surface. It has a reflectance maximum near 560 nm.^70–72^ A stable colored complex was formed after 10 min, as indicated by the constant reflectance intensity. At reaction times of less than 10 min, complex formation (Cr­(III)–DPCO) on the solid surface was incomplete. Beyond 10 min, the reflectance intensity of the solid phase remained nearly constant, suggesting that an equilibrium had been reached.

#### 3.2.6 Elution of the Colored Complex from the Nanoparticle Surface

The effect of eluent type on the desorption efficiency of Cr­(VI)–DPC complexes was examined using various eluents, including HCl, HNO_3_, CH_3_COOH, H_2_SO_4_, acetone, ethanol, *N*,*N*-dimethylformamide (DMF), methanol, and their combinations (Figure 6F). Different elution agents were evaluated to determine their effectiveness in desorbing Cr­(VI)–DPC complexes (i.e., Cr­(III)–DPCO) from the adsorbent surface. It has been established that the cationic Cr­(III)–DPCO complex is formed via the redox reaction between DPC and Cr­(VI), as shown in eq 3. This mechanism is hypothesized to proceed through nascent, nonhydrated Cr­(III) ions newly formed Cr­(III) generated during the reduction of Cr­(VI).^71,73^ Quantitative desorption of Cr­(VI)–DPC from the PDDA@PWA adsorbent was achieved only with the LBR@DMF mixture (1:1). Considering that the adsorbent required sufficient eluent to ensure complete desorption, four volumes of 200, 300, 400, and 500 μL were tested by using the LBR@DMF mixture as the desorption solvent (Figure 6G). The best desorption was performed using 400 μL of LBR@DMF for 5 min. Furthermore, the desorption time was carefully optimized for different time intervals (1.0–10 min) with 400 μL of elution solution. The maximum desorption efficiency was achieved within 5 min (Figure 6H). During the elution, the mixture was gently shaken by hand for 5 min to ensure thorough mixing of the eluent and adsorbent, thereby promoting mass transfer and enhancing the desorption efficiency. In this case, as shown in Figure 7A, the PDAA@PWA returns to its initial white solid form. As a result, the remaining solid material can be reused in another cycle of Cr­(VI) adsorption/desorption. Figure 7A illustrates the schematized method for the desorption of PDDA@PWA after Cr­(VI) adsorption. In elution systems, DMF’s unique polarity helps disrupt adsorbent–analyte interactions, weakening noncovalent forces such as hydrogen bonding or van der Waals interactions between the analyte and adsorbent surface. Consequently, analyte desorption or recovery is facilitated, leading to enhanced analytical performance.^74^ It was observed that increasing the volume of the extraction phase led to a larger total volume of LBR@DMF but resulted in a noticeable decrease in the extraction efficiency. Therefore, 400 μL of LBR@DMF was determined to be the optimal elution volume.

3 $$2{{\mathrm{CrO}}_{4}}^{2-}+3C_{13}H_{10}{\mathrm{ON}}_{4}H_{4}+8H^{+}\to {\left[\mathrm{Cr}{\left(C_{13}H_{10}{\mathrm{ON}}_{4}H\right)}_{2}\right]}^{+}+{\mathrm{Cr}}^{3+}+C_{13}H_{12}{\mathrm{ON}}_{4}H_{2}+8H_{2}O$$

**Figure 7:**
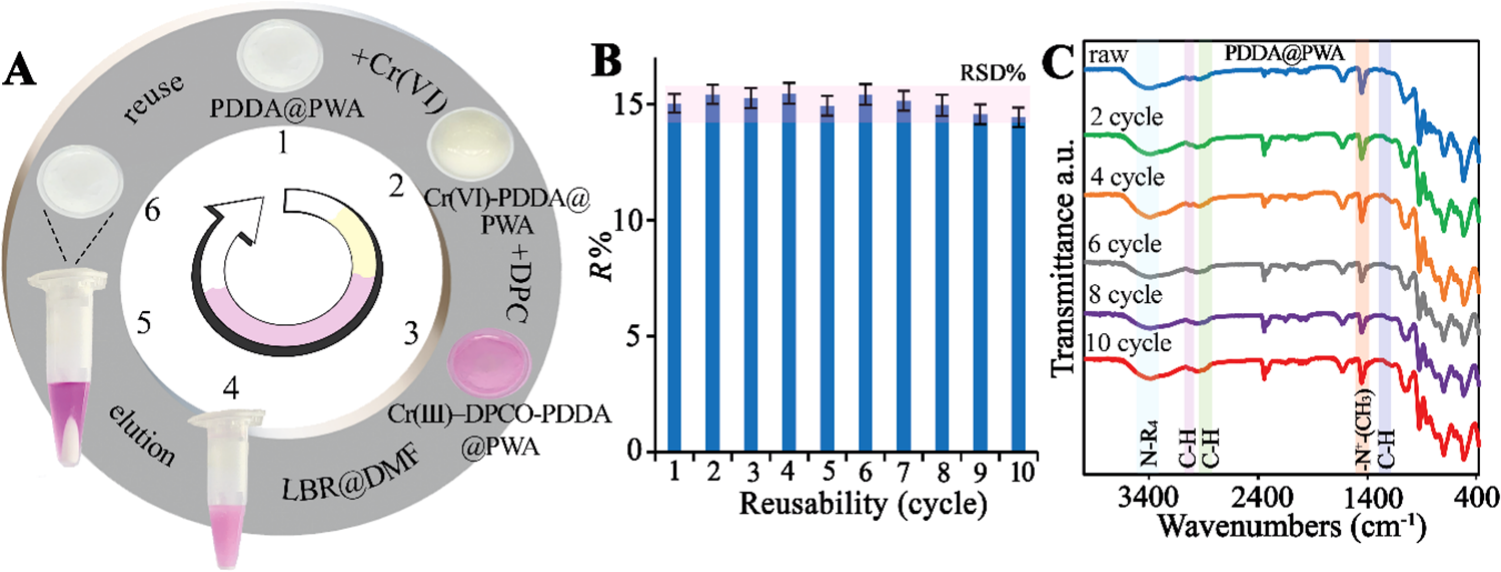
(A) Evaluation of Cr­(VI) adsorption–desorption efficiency and adsorbent regeneration using LBR@DMF as the eluent. (B) Reusability performance of the PDDA@PWA nanoparticles over ten consecutive adsorption–desorption cycles. (C) Comparison of FTIR spectra of PDDA@PWA before (raw material) and after adsorption/desorption cycles (2, 4, 6, 8, and 10).

### 3.3 Reusability of the Adsorbent

Reusability is a key parameter for assessing the practicality and performance of an adsorbent in real-world applications. To evaluate the reusability of the PDDA@PWA composite, ten consecutive adsorption–desorption cycles were conducted under optimal conditions using the same 15 mg of adsorbent (Figure 7B). The optimal desorption parameters were determined to be 400 μL of LBR@DMF as the eluent and a desorption time of 5 min. The adsorbent was regenerated by eluting the Cr­(VI)-DPC complexes using the LBR@DMF solution, followed by washing and neutralization to return it to its initial state. The reflective intensity of the target analytes remained nearly constant throughout the ten cycles, with RSDs ranging from 4.9% to 5.2%. These results demonstrate that the PDDA@PWA composite exhibits excellent reusability and operational stability.

### 3.4 Effect of Desorbing Agent on Adsorbent Characteristics

Adsorbent materials may decompose, dissolve, or aggregate in desorption reagents due to chemical instability or harsh regeneration conditions such as extreme temperatures and pH levels. This structural degradation leads to a loss of surface area and leaching from the support, significantly reducing their reusability in catalysis and adsorption processes. To investigate the evolution of surface functional groups and to evaluate structural stability, FTIR spectra were obtained for the raw material (15 mg) and the adsorbent after multiple adsorption–desorption cycles. As shown in Figure 7C, FTIR spectra were obtained following 2, 4, 6, 8, and 10 adsorption–desorption cycles. The adsorbent exhibited remarkable chemical robustness, as evidenced by the unchanged FTIR spectra after multiple adsorption–desorption cycles. The composite also exhibited excellent cycling stability. The adsorbent was effectively regenerated using LBR@DMF; notably, the analyte’s reflectance intensity retained 13.90% of its initial value after ten adsorption–desorption cycles. Ultimately, the eluent used (LBR@DMF) does not damage the adsorbent.

### 3.5 Analytical Characteristics

Following sensing studies, analytical data were obtained by SPE of the Cr­(VI) species on the PDDA@PWA platform by using a batch process. The reflectance colorimetric assay was performed directly on the Cr­(VI)-enriched PDDA@PWA surface. The *R*% values of the solid phase were obtained through FORS. Figure 8A displays the effect of the Cr­(VI) concentration on the *R*% values. The relative reflectance (*R*%) values of the peak current increased as the concentration of Cr­(VI) increased. Thus, the reflectance peak points are plotted against the corresponding concentrations of Cr­(VI) in Figure 8A. The obtained *R*% is established linearly in the Cr­(VI) concentration range of 0.2–10 μM, which is denoted as the detection range of Cr­(VI). The linear regression equation was *R*% = 7.0659 C (μM) + 1.0042 (*R* ^2^ = 0.9968). The results demonstrate that direct modeling of *R*% offers requisite accuracy, rendering the Kubelka–Munk (K–M) remission function unnecessary. As a result, the sensitivity of the approach is below the Cr­(VI) concentration of 0.05 mg/L reported by the WHO in drinking water. As can be seen, increasing *R*% intensity related to the concentration of the analyte could be visibly monitored from the solid-phase images. The research results showed that images are suitable for use in estimating the analyte concentration in solid phases. The naked-eye readable colors corresponding to the concentrations of the Cr­(VI) ions (0.2–10.0 μM) are photographed in Figure 8D.

**Figure 8:**
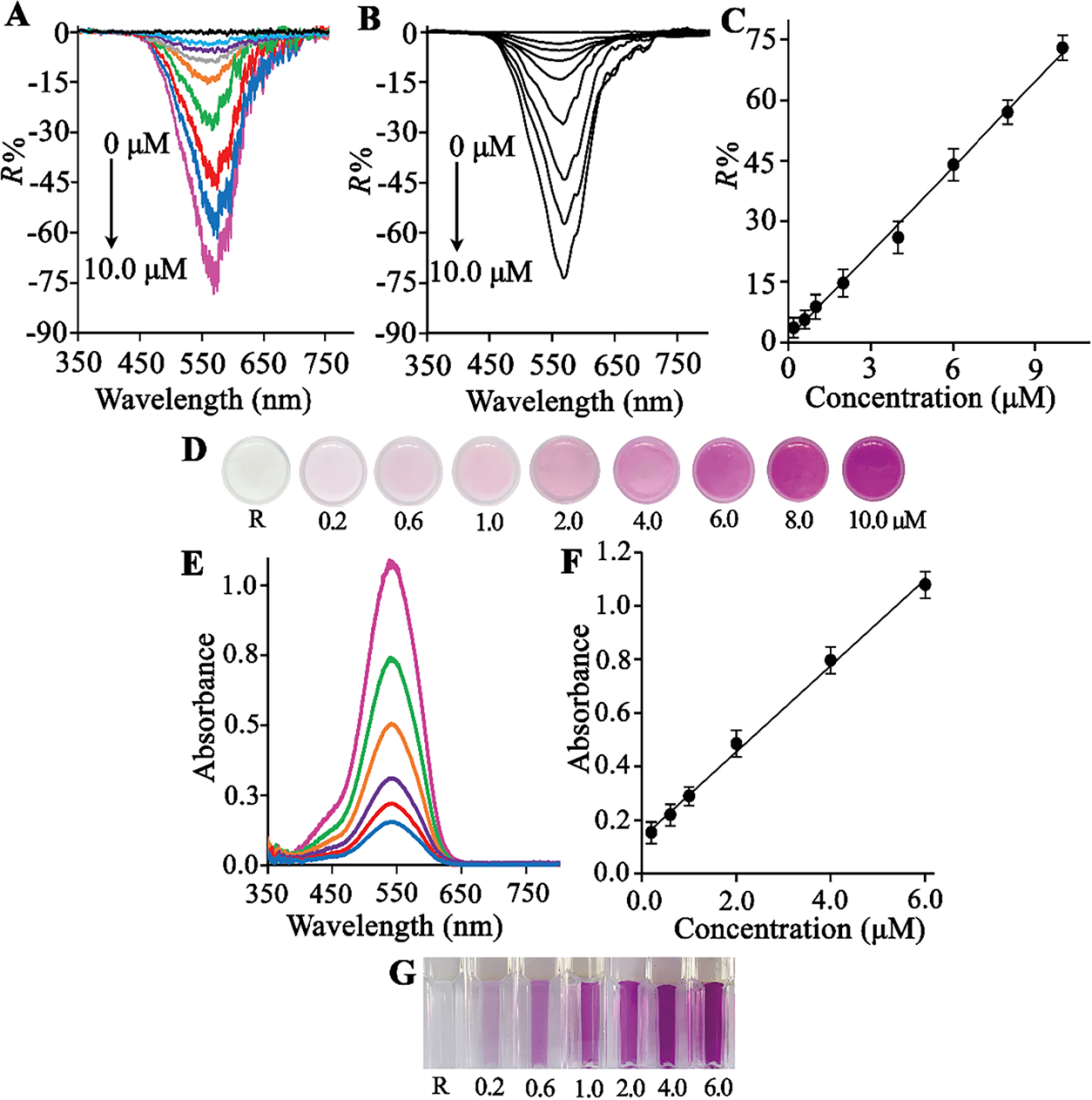
(A) Reflectance spectra of different Cr­(VI) concentrations, (B) smoothed reflectance spectra different Cr­(VI) concentrations, (C) calibration curve (D) concentration assignment for visual assay, (E) FO–UV–vis spectra of different Cr­(VI) concentrations after elution, (F) a linear calibration curve, (G) concentration assignment for visual assay. SPE conditions: sample pH: 4.0, sorbent amount: 15 mg, adsorption time: 20 min, elution time: 15 min, elution volume: 400 μL of LBR@DMF.

Subsequently, the Cr­(VI) complex collected on the PDDA@PWA NPs was readily eluted with 400 μL of the eluent within 5 min. The resulting eluate was then analyzed via fiber-optic UV–vis spectrophotometry (FO–UV–vis), allowing for a direct comparison between the solid-state reflectance data and traditional absorbance measurements. The absorbance of the eluent obtained was constant for at least 60 min. The calibration curve obtained by the standard procedure was linear over the concentration range of 0.2–6.0 μM Cr­(VI) in 400 μL of the eluent (LBR@DMF). The apparent molar absorptivity was 1.6 × 10^5^ dm^3^ mol^–1^ cm^–1^ at 541 nm. The relative standard deviation was 2.7% for 1.0 μM Cr­(VI) (*n* = 3). A linear regression equation and correlation coefficient were *A* = 0.1608 C (μM) + 0.1334 and *R* ^2^ = 0.9970 (*n* = 3), respectively, where *A* is the absorbance at λ = 540 nm, and *C* is the concentration of the analyte in μM. Figure 8E shows the spectra of Cr­(VI) in the eluents, and Figure 8F shows the corresponding calibration curve. The naked-eye readable colors corresponding to the concentrations of the Cr­(VI) ions (0.2–6.0 μM) are photographed in Figure 8G. The detection limit (LOD = 3Sb/m) was estimated as 0.01 μM. As the analyte in 10 mL of the sample solution was preconcentrated into 400 μL, the preconcentration factor was estimated to be 40. The relative standard deviations (RSD) for 0.5 and 1.0 μM Cr­(VI) were 4.86% and 3.86% (for *n* = 3), respectively. The molar absorption coefficient (ε) is equal to 1.6 × 10^5^ L mol^–1^ cm^–1^ (without preconcentration, ε is equal to 4.22 × 10^–4^ L mol^–1^ cm^–1^).

### 3.6 Effect of Foreign Ions

The goal was to test how other ions or substances might interfere with the detection of Cr­(VI) (at 2.0 μM) under the SPE conditions optimized earlier in the study. Any species causing more than a 5% change in reflectance intensity was considered an interfering substance. Common metal ions (Na^+^, K^+^, Ca^2+^, Mg^2+^, Cr^3+^, Zn^2+^, Cd^2+^, Co^2+^, Cu^2+^, Al^3+^, Ni^2+^, Mn^2+^, Ba^2+^, Fe^3+^) did not affect the reflectance intensity of the Cr­(VI) signal, even when present at 100 times the concentration of Cr­(VI). Hg^2+^ also showed no interference, even in a 100-fold excess. As^3+^, however, produced a noticeable decrease in the intensity of the Cr­(VI) signal even at a 10-fold excess, indicating partial interference. According to the above examinations, the obtained *R*% values were within the ranges of 14.90% and 14.45%. The PDDA@PWA platform repels other positive cations due to charge similarity, which reduces interference from metal cations. Since the water sample typically contains several inorganic anions that may potentially interfere with the binding between Cr­(VI) and PDDA@PWA, it is indispensable to examine the effect of other interfering ions on the retention or removal process of Cr­(VI). As shown in Figure 9B, the inorganic anions (10-fold mol ratios) such as NO_3_-, SO_4_^2–^, and PO_4_^3–^ were found to have an insignificant effect on the adsorption performance of PDDA@PWA. The obtained *R*% was within the range of 14.90% and 14.45%. When mol ratios of competing anions were increased further to 100-fold, the reflectance intensity (*R*% = 15.12 ± 0.07) decreased to 13.05 ± 0.06% (NO_3_^–^), 12.15 ± 0.08% (SO_4_^2–^), and 12.11 ± 0.05% (H_2_PO_4_^–^). Additionally, the calculated percentage recoveries were found to be 86.34%, 80.36%, and 80.10%, respectively. As PDDA is a positively charged polymer, its anion retention mechanism is primarily driven by electrostatic attraction. PWA, on the other hand, provides structural stability and offers specific binding sites within the composite. If the presence of ions like nitrate, sulfate, and phosphate significantly reduces efficiency, this indicates that the binding is dominated by nonspecific electrostatic interactions. Common organic compounds such as glucose and several carboxylic acids (lactic, malic, tartaric, oxalic, and citric acids) also showed no interference, even at 100 times the concentration of Cr­(VI). The PDDA@PWA platform provides a highly selective detection of Cr­(VI), with minimal interference from most ions and organic compounds typically present in water. In summary, this study demonstrates that the developed reflectance colorimetric detection system for Cr­(VI) is selective, stable, and accurate under realistic conditions with only minor susceptibility to As­(III). It can be seen from Figure 9 that all the reflectance intensities were close to that of the blank signal.

**Figure 9:**
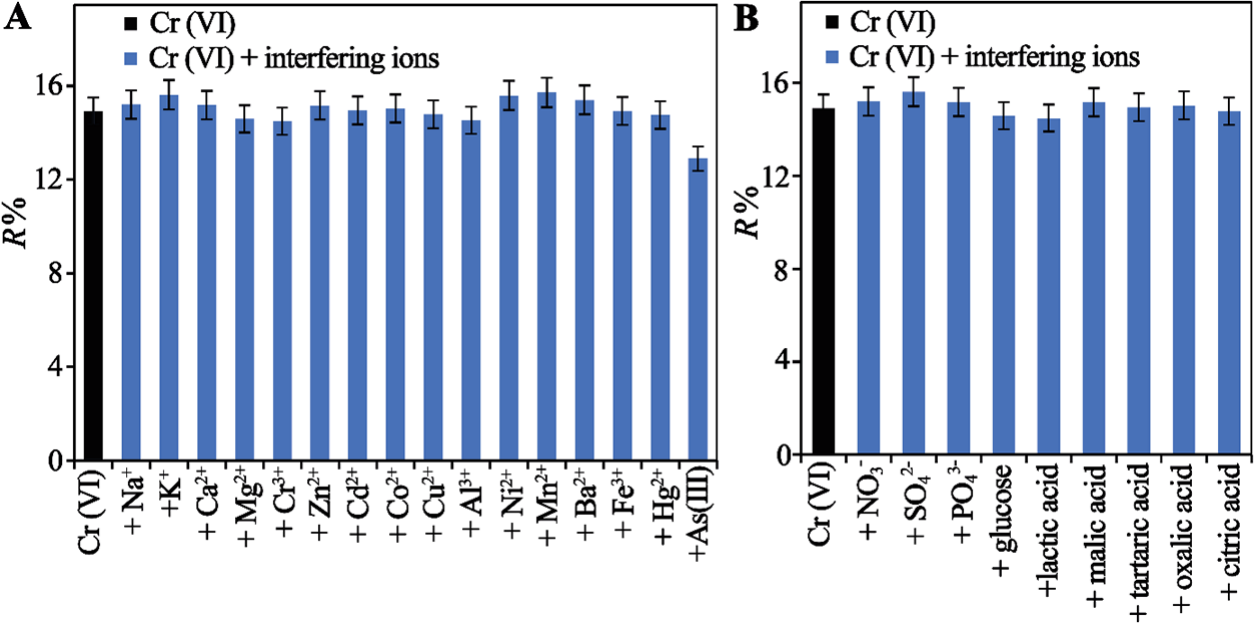
Selectivity of PDDA@PWA (2.0 μM) toward (A) cations and (B) anions, and organic molecules.

### 3.7 Method Comparison

In this study, the analytical performance of the proposed reflectance colorimetric method was compared with that of previously reported techniques (Table 1). The reflectance-based colorimetric method developed here exhibited superior^3,19,20,23^ or comparable^22,24^ analytical capabilities to other adsorptive colorimetric methods, particularly in terms of the limit of detection (LOD). Notably, this work introduces PDDA@PWA NPs as a novel material for the simultaneous SPE and FORS determination of Cr­(VI). To the best of our knowledge, reflectance-based colorimetry has not previously been applied to Cr­(VI) detection, as summarized in Table 1. Furthermore, the PDDA@PWA-based adsorbent demonstrated excellent reusability over more than ten regeneration cycles, indicating its potential for cost-effective and sustainable commercial applications. Overall, the proposed approach offers a robust, sensitive, and efficient alternative for the detection of toxic Cr­(VI) ions in aqueous matrices.

**Table 1:** Comparison of the Proposed Method with Other Methods

	UV–vis spectrophotometric method		adsorptive colorimetric method		
sorbent	linear range (μg/L)	LOD (μg/L)	linear range (μg/L)	LOD (μg/L)	refs
cationic waste cotton fabric	-	-	10–1000	13	^3^
amine-functionalized silica	-	-	500–10000	500	^19^
cellulose-based solid amine	-	-	NR	500	^20^
imidazolium-polymer^a^	-	-	0.26–910	0.092	^21^
amberlite XAD-7HP	-	-	2.0–100	3.4	^22^
DPC-alginate/pectin	-	-	2000–10000	233	^23^
microfluidic paper	-	-	7.0–200	2.0	^24^
PDDA@PWA NPs	10.4–312	0.52	10.4–520	1.82	this work

a Fluorimetric/colorimetric assay.

### 3.8 Real Sample Analysis

To assess the practical applicability of the proposed FORS method, a tap water sample collected from the University Campus (Istanbul/Turkey) was analyzed. Before analysis, the tap water samples were first filtered using standard filter paper and then spiked with known concentrations of Cr­(VI) at four levels (0.6, 0.8, 1.0, and 2.0 μM). Each concentration level was analyzed in triplicate (*n* = 3) using the standard addition technique to account for possible matrix effects. A good correlation was observed between the measured and added analyte concentrations, indicating reliable quantification. As summarized in Table 2, the mean recoveries of Cr­(VI) in the spiked samples ranged from 93% to 97%, with relative standard deviations (RSD) below 5%. These results confirm the high accuracy and precision of the proposed method and demonstrate that the determination of Cr­(VI) is not significantly affected by the sample matrix. The method shows strong recovery (93–97%) from real water samples, confirming its reliability and robustness. Furthermore, following the elution of the Cr­(VI)–DPC complexes, reflectance colorimetric results obtained by using the FORS technique showed excellent agreement with FO–UV–vis spectrophotometric measurements, further supporting the validity and robustness of the method. The results obtained are summarized in Table 2. The Cr­(VI) concentration calculated from the eluate matches the Cr­(VI) concentration measured by FORS, demonstrating that surface-based sensing is reliable. Moreover, the accuracy of the methods was assessed by the analysis of the CRM. The result shows that the obtained value is (426.7 ± 15.5 μg/L) consistent with the reported value (438 ± 4.15 μg/L), verifying the accuracy of the FORS method. All results indicate that the FORS-based platform is feasible for rapid, convenient, and accurate estimation of the Cr­(VI) residue in actual samples.

**Table 2:** Analysis of Cr­(VI) in Spiked Water Samples Using PDDA@PWA NPs with FO–UV–Vis Spectrophotometric and FORS-Based Colorimetric Method

		FO–UV–vis spectrophotometry (*n* = 3)		FO-reflectance colorimetry (*n* = 3)	
sample	added (μM)	found (μM)	recovery (%)	found (μM)	recovery (%)
water		<LOD		<LOD	
	0.6	0.58 ± 0.02	96.67	0.56 ± 0.03	93.33
	0.8	0.78 ± 0.03	97.50	0.76 ± 0.04	95.00
	1.0	0.98 ± 0.03	98.00	0.96 ± 0.05	96.00
	2.0	1.97 ± 0.08	98.50	1.95 ± 0.08	96.50

## 4 Conclusions

This study reports a solid-phase extraction method for the reflectance colorimetric determination of Cr­(VI) in aqueous samples using PDDA@PWA nanocomposite adsorbents. The proposed FORS-based method demonstrates a linear response over the concentration range of 0.2–10 μM, with a detection limit of 0.035 μM. The PDDA@PWA nanoparticles are recyclable and can be reused multiple times, representing a sustainable and cost-effective platform for toxic anion sensing. The system enables real-time and on-site detection of Cr­(VI), making it particularly suitable for portable applications. The adsorbent can be efficiently regenerated using an LBR@DMF (1:1) treatment mixture and reused for at least ten consecutive cycles without a significant loss of performance. The positively charged amino functionalities grafted onto PWA afford superior adsorption capacity, selectivity, and stable retention of Cr­(VI) even in the presence of potential interferences. Overall, the PDDA@PWA platform offers an accurate, efficient, and affordable approach for Cr­(VI) quantification without the requirement of labor-intensive intermediate steps. Its advantages, including high sensitivity, rapid detection, low energy demands, and operational simplicity, make it especially valuable in resource-limited settings. In addition, the methodological framework developed herein allows for concurrent matrix isolation, preconcentration, and chromium speciation, providing a versatile foundation for future analytical extensions and applications in environmental monitoring.
